# The contribution of molecular epidemiology to the understanding and control of viral diseases of salmonid aquaculture

**DOI:** 10.1186/1297-9716-42-56

**Published:** 2011-04-05

**Authors:** Michael Snow

**Affiliations:** 1 Marine Scotland Science, 375 Victoria Road, Aberdeen, AB11 9DB Scotland, UK

## Abstract

Molecular epidemiology is a science which utilizes molecular biology to define the distribution of disease in a population (descriptive epidemiology) and relies heavily on integration of traditional (or analytical) epidemiological approaches to identify the etiological determinants of this distribution. The study of viral pathogens of aquaculture has provided many exciting opportunities to apply such tools. This review considers the extent to which molecular epidemiological studies have contributed to better understanding and control of disease in aquaculture, drawing on examples of viral diseases of salmonid fish of commercial significance including viral haemorrhagic septicaemia virus (VHSV), salmonid alphavirus (SAV) and infectious salmon anaemia virus (ISAV). Significant outcomes of molecular epidemiological studies include:

Improved taxonomic classification of viruses

A better understanding of the natural distribution of viruses

An improved understanding of the origins of viral pathogens in aquaculture

An improved understanding of the risks of translocation of pathogens outwith their natural host range

An increased ability to trace the source of new disease outbreaks

Development of a basis for ensuring development of appropriate diagnostic tools

An ability to classify isolates and thus target future research aimed at better understanding biological function

While molecular epidemiological studies have no doubt already made a significant contribution in these areas, the advent of new technologies such as pyrosequencing heralds a quantum leap in the ability to generate descriptive molecular sequence data. The ability of molecular epidemiology to fulfil its potential to translate complex disease pathways into relevant fish health policy is thus unlikely to be limited by the generation of descriptive molecular markers. More likely, full realisation of the potential to better explain viral transmission pathways will be dependent on the ability to assimilate and analyse knowledge from a range of more traditional information sources. The development of methods to systematically record and share such epidemiologically important information thus represents a major challenge for fish health professionals in making the best future use of molecular data in supporting fish health policy and disease control.

## Introduction

### What is molecular epidemiology?

**Figure 1:**
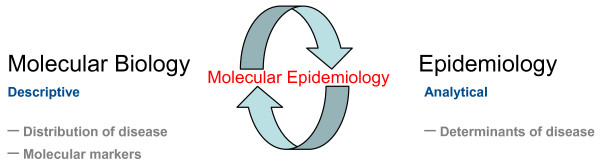
**Definition of molecular epidemiology, spanning the disciplines of descriptive and analytical epidemiology**.

Epidemiology, or the study of factors affecting the health of populations is as old as science itself, with Hippocrates (460-377 BC) being the first to examine the relationship between disease occurrence and environmental issues. More recent technological developments have facilitated the identification and exploitation of molecular biomarkers, whose use alongside traditional epidemiological approaches has led to a better understanding of the underlying mechanisms of disease transmission in populations. This young science of "molecular epidemiology" has emerged since the 1970s when the term was first coined in relation to the study of influenza virus [1]. Molecular epidemiology utilises molecular biology to define the distribution of disease in a population (descriptive epidemiology) but still relies heavily on integration of traditional (or analytical) epidemiological approaches to identify the etiological determinants of such relationships (Figure 1).

Descriptive molecular epidemiology most often involves an attempt at establishing the evolutionary history of a given viral species called its phylogeny. A phylogenetic tree is a graphical summary of this inferred evolutionary relationship between isolates from which the pattern and in some cases timing of events that occurred as viruses diversified can be hypothesised. Since all life is related by common ancestry, viruses (or other organisms) in current circulation display genetic diversity which reflects their evolutionary history due to the accumulation and inheritance of mutations when genetic material is copied. In the absence of historical information or isolates, which are often unavailable, the evolutionary history of viruses in current circulation can be hypothesised based on the sampling of current genetic markers and by working backwards to infer the most likely series of events which best explain the observed relationships. Fundamental to reconstructing such an evolutionary history is the comparison of homologous characters (often nucleotide or amino acid sequence positions); i.e. those which descend from a common ancestor. In practice, this involves creating an alignment of sequences which represents a hypothesis of positional homology and provides the basis for reconstructing evolutionary history based on a mathematical model of evolution. This review aims to focus on the contribution that the application of such descriptive tools has made to the practical understanding and control of viruses of salmonid aquaculture, rather than the techniques themselves, which have been extensively reviewed by others (e.g. [2]).

Analytical epidemiology is based on observations of disease trends and incidence in different populations that turn into testable hypotheses, and entails rigorous collection of data for all aspects of study [3]. As noted by Porta et al. [4], the lack of careful application of the science of epidemiology can limit the amount of useful information obtained from studies of molecular epidemiology. This remains a significant challenge in the field of fish virology where the application of descriptive molecular epidemiology is gathering momentum, yet data required for adequate interpretation is often lacking or at best collected haphazardly. Despite this, molecular epidemiology has made a significant contribution to the understanding and control of viral diseases of fish. This review aims to illustrate some of these contributions, whilst highlighting some future challenges faced in this field to ensure that molecular epidemiology fulfils its potential of converting complex viral transmission pathways into relevant fish virus control policies.

### Setting the scene: fish viruses, aquaculture and molecular epidemiology

Fish, unlike many other domestically reared farmed animals, are most often reared in open systems (cages) where they are exposed to a wide diversity of naturally occurring environmental pathogens. Recent estimates suggest the presence of a staggering 10^8 ^viruses/mL in coastal seawater [5]. Although the majority of these viruses appear to be infective [6], most are pathogens of planktonic organisms and have a narrow range of potential host species [5]. Whilst the importance of the role of viruses as agents of microbial mortality and regulators of global carbon and nutrient cycling in the oceans is being realised [7], some viruses are also, clearly, progenitors of disease in higher organisms such as fish. Given the extent of the viral genetic diversity present in both marine and freshwaters [8], it is perhaps not surprising that viruses of fish are represented in most of the major established viral genera (for review see [9]). Indeed, culturing fish in an open seawater (or freshwater) system rich in viral diversity clearly presents opportunity for the introduction of many potential pathogens into aquaculture systems. The good news is that the vast majority of these potential progenitors of disease do not cause a problem in healthy wild and farmed fish populations. In natural systems, where viruses and their piscine hosts have apparently existed for millenia, this would appear to be a consequence of co-evolution, where killing of the host might otherwise have led to viral extinction. In support of this, examples exist of naturally occurring marine reservoirs of fish viruses recovered from asymptomatic hosts with no demonstrated deleterious effect on fish populations [10-12]. However virulence can still be an adaptive response of pathogens [13], and disease can be a significant factor in regulating natural fish populations [14,15].

The advent of intensive fish husbandry and associated international movements of fish has fundamentally altered the natural equilibrium by exposing animals to new environments and their viruses to new host species [16]. As a consequence, increased rates of evolution of fish viruses are apparent in aquaculture as compared to those in natural systems [17]. In intensive culture systems, higher rates of evolution result from increased selective pressures from factors associated with modern aquaculture including the use of novel host species, continuous addition of susceptible fish, high rearing densities, differing temperatures and the presence of immunised fish [18]. The effects of such selection pressures are further amplified by virtue of the fact that many significant viral pathogens of fish have an RNA genome. Viruses with RNA genomes are characterised by the presence of an error-prone RNA polymerase and high mutation rates. This results in the generation of "quasispecies" populations comprised of a spectrum of mutant genomes which are continually generated [19]. This constitutes the raw material for evolution, with such mutants potentially having a greater biological fitness, thus providing a mechanism for adaptation to changing conditions.

The accumulation of relatively high levels of genetic variation in viruses associated with aquaculture provides excellent and exciting opportunities for understanding the relative contribution of molecular epidemiology to their understanding and control. In order to identify the extent to which this capacity has been exploited in this field, this review focuses on three of the most significant and well studied RNA pathogens of salmonid aquaculture, viral haemorrhagic septicaemia virus (VHSV), infectious salmon anaemia virus (ISAV) and salmonid alphavirus (SAV). In this area the application of molecular epidemiology has provided important new knowledge of fundamental importance to disease management and control in the following key areas:

• Viral taxonomy and classification

• Understanding origins of diseases of aquaculture

• Viral phylogeography and risks associated with viral translocation

• Outbreak tracing and disease control

• Genetic diversity and viral surveillance

## Case studies highlighting the contribution of molecular epidemiology to the understanding and control of viral disease of salmonid aquaculture

### Viral haemorrhagic septicaemia virus

#### Overview

Viral haemorrhagic septicaemia (VHS) was, until the late 1980s thought to be a disease exclusive to the freshwater rainbow trout industry of Continental Europe, which is characterised by extensive degeneration and necrosis of the internal tissues [20]. The disease can result in mortalities of up to 90% [21]. Multiple occurrences of disease were later associated with European [22,23] and Asian [24] marine aquaculture and a widespread global marine distribution of VHS virus in an ever increasing variety of largely aysmptomatic marine host species is now acknowledged. A growing body of evidence suggests the original import of VHSV into freshwater systems from marine sources, with the most recent example of this being the apparent introduction of VHSV into the Great Lakes system of North America where extensive mortality in wild fish has resulted [25,26].

#### Molecular epidemiology facilitates the taxonomic classification of VHSV and provides a basis for understanding the relationship between isolates

VHSV is one of several important fish viral species that were originally identified as belonging to the family *Rhabdoviridae *based on the distinctive morphology of viral particles in electron micrographs. Until relatively recently, insufficient data existed to assign any of the fish rhabdoviruses to novel genera with tentative groupings of "unassigned" and "vesiculovirus-like" viruses being proposed based on protein electrophoretic data and antigenic relatedness [27]. Sequence determination and phylogenetic analysis of the glycoprotein gene subsequently indicated that the lyssavirus-like viruses, including VHSV, infectious haematopoietic necrosis virus (IHNV) and hirame rhabdovirus (HIRRV) warranted classification within a new genus [28]. This distinction has since been supported by the comparison of genetic variation at the G-L gene junction which represents a major point of distinction between the different established genera of rhabdoviruses [29]. Indeed, the presence of an additional and characteristic NV gene at this junction in the fish viruses VHSV, IHNV and HIRRV supported their designation within a new genus (genus *novirhabdovirus*) [30]. Taxonomic classification of fish viruses is important in identifying their potential biological properties based on comparison with their often better studied counterparts (e.g. rabies virus). Such studies have also represented the first steps towards a deeper understanding of the classification of fish rhabdoviruses such as VHSV which have led to significant and tangible outcomes of practical relevance to disease control.

**Figure 2:**
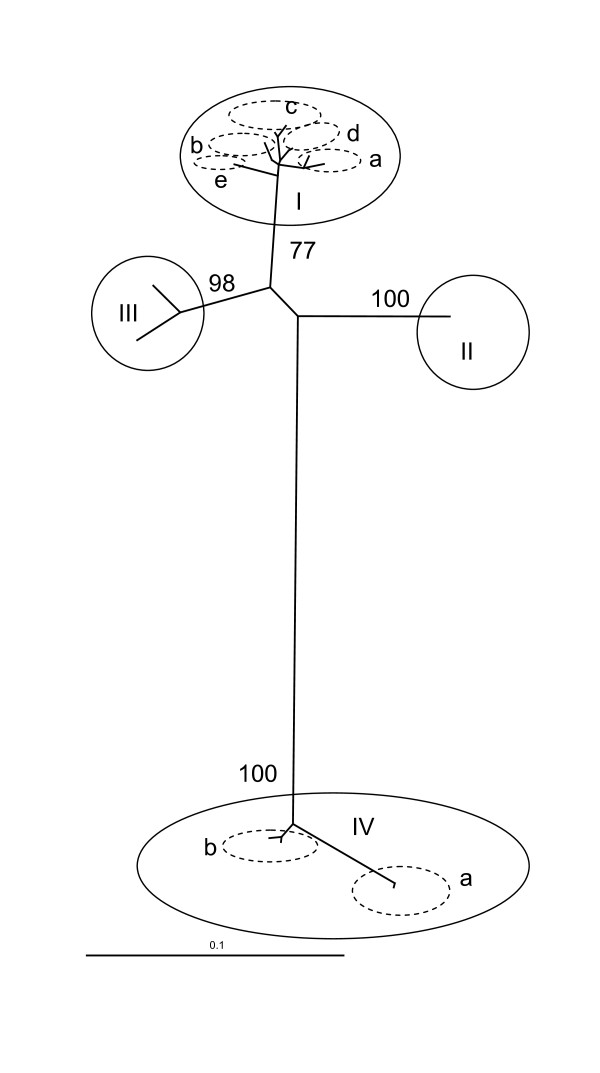
**Overview of the genetic relationships between the main recognized genotypes of VHSV based on a dataset comprised of partial G gene data from 2 representatives of each of the established major genetic subgroups**. Publicly available sequences derived from up to 2 isolates from each genotype recognized to date were imported into Bioedit version 7.0.5.3 [76] and a multiple alignment performed using Clustal X [77]. The final alignment consisted of 17 unique sequences spanning a partial region of the VHSV G Gene (649nt). The phylogenetic relationship among VHSV isolates was inferred using a maximum likelihood based approach implemented within PAUP* (version 4.0; [78]) and using the PaupUP interface v1.0.3.1 [79]. The jModeltest 0.1.1 program [80]) was used to identify the model that best fits the sequence data from 56 models using the Akaike Information Criterion (AIC; [81]). The optimal unrooted maximum likelihood tree was identified using a heuristic search implemented in PAUP* and evaluated using 100 bootstrap iterations [82]. Significant bootstrap values for the major clades were transferred to the unrooted tree derived from the original data.

To date, four main genetic groups of VHSV are recognised worldwide [17,31], as depicted in Figure 2. In the case of VHSV, these genotypes reflect a largely geographic rather than a species-specific distribution and in the absence of recombination in negative strand viruses have been shown to be robust and independent of the preferred gene of analysis [32]. Genotype I includes a wide range of viruses originating from freshwater rainbow trout farms in continental Europe [17,33], and a large number of isolates originating from marine species in the Baltic Sea/Skagerrak/Kattegat/English channel [17,31,34]. These isolates fall within 2 of 5 proposed subgroups within Genotype I (subgroups 1a and 1b respectively) [17]. Marine isolates from subgroup 1b differ markedly from other Genotype I isolates since it has been experimentally shown that they are of low pathogenicity to rainbow trout [35]. The remaining subgroups identified within Genotype I include isolates recovered from Danish freshwater rainbow trout farms in the 1980s (subgroup Ic), marine-farmed rainbow trout in Scandinavia (subgroup Id) [17,36] and isolates recovered from turbot and rainbow trout in Georgia and the Black Sea region (subgroup Ie) [37]. Genotype II includes a limited number of VHSV isolates recovered from the Baltic sea [31], while Genotype III includes isolates originating from outbreaks of VHS in turbot farms in the British Isles along with a number of isolates recovered from a variety of marine species caught in Scottish and North Atlantic waters [31]. The final genetic group of VHSV isolates identified to date comprises a range of isolates recovered from wild marine fish from the Pacific Northwest [24,38,39] and eastern coastal areas of Canada and the USA, isolates from Japanese flounder [24] and isolates originating from a recent series of epidemics in the Great Lakes of North America [25,26]. Establishing the genetic relationships between currently circulating isolates of VHSV through molecular epidemiological studies has led to new understanding in a number of areas of critical importance to disease control and the development of sustainable aquaculture.

#### Understanding genetic relationships provides an insight into the origins of VHS in aquaculture

VHS was traditionally thought to be a disease exclusive to freshwater rainbow trout farming, where it has historically been responsible for significant economic loss. The first and subsequent identification of marine isolates of VHSV virus of differing genetic type in Europe [40] and North America [41] questioned this dogma. The fact that these genotypes were indicated to have become separated a long time before fish farming was established on both continents [28], coupled to the fact that VHS is not a disease problem in rainbow trout in North America, indicated existence of long standing natural marine reservoirs of VHS virus. The existence of naturally occurring marine reservoirs of VHS virus is now widely recognised and a long list of species which may be infected with the virus has been established (an updated list of natural and experimental hosts for VHSV can be found via the International Database on Aquatic Animal Disease [42]). The demonstration that some of these naturally occurring marine VHS viruses (Genotype 1b; Figure 2) bear a close genetic relationship to those responsible for disease in freshwater rainbow trout farms (Genotype 1a; Figure 2) suggested that such isolates represent the most likely origins of introduction of VHS into rainbow trout aquaculture in Europe (for a review see Skall et al. [43]). Since the use of unpasteurised "trash" marine fish was widely used as a source of aquaculture feed in the early days of the industry, this may be a mechanism via which the pathogen was introduced. Whatever the mechanism, since naturally occurring marine genotype 1b viruses have been shown to be non-pathogenic for rainbow trout [35], it is clear that the virus must have adapted a new pathogenic life-strategy within rainbow trout aquaculture. This may be a consequence of the high selective pressures and continuous availability of susceptible hosts associated with aquaculture. Such conditions may favour development of an alternate "pathogenic" life-strategy to that in the natural environment where death of the host may represent an evolutionary dead-end. The introduction of VHSV from the marine environment into rainbow trout aquaculture appears to have occurred on more than one occasion. Indeed, the identification of Genotypes 1c, 1d and 1e [17] has led to the interpretation that host adaptation from marine environment/species to rainbow trout has occurred 3 or 4 times in freshwater farms in Denmark and more recently in fish reared at the coast of Finland and Sweden [17].

Until recently, only the adaptation of marine Genotype I strains of VHSV in rainbow trout had been shown to occur. Much like the Genotype Ib marine isolates, other naturally occurring genotypes of VHSV have been experimentally shown to be of low virulence to rainbow trout [35]. The recent demonstration that VHS disease in a marine rainbow trout farm in Norway was caused by a Genotype III virus [44], suggests that the potential for adaptation to this species is not an exclusive property of Genotype I viruses. Indeed, given the potential for continuous adaptation of RNA viruses, it is perhaps not surprising to find that other viruses are capable of dramatic shifts in host specificity given the appropriate opportunity. Such opportunity in an aquaculture context can include factors such as the use of continuous production systems without synchronous fallowing which can allow pathogens to persist for long periods resulting in increased potential for adaptation to occur.

While the above examples assume the input and subsequent adaptation of naturally non-pathogenic viruses in aquaculture systems, naturally occurring viruses can in some cases also cause disease without requirement for change. Following outbreaks of disease in marine farmed turbot in the British Isles caused by VHSV [22], experimental trials with a range of naturally occurring GIII isolates indicated that turbot were susceptible to these viruses [45]. The susceptibility of turbot to naturally occurring VHSV is thus a significant risk to the sustainable production of turbot within the natural range of such isolates. The susceptibility of turbot to naturally occurring VHSV is at odds with the conventional wisdom that pathogens evolve toward non-pathogenic lifestyles in natural systems. This may be a consequence of the lack of exposure to the pathogen in nature with commercial aquaculture resulting in the exposure of previously naïve species to new pathogens. Another example of a pathogen which can cause disease without adaptation is that of VHS disease epidemics which occur periodically in wild Pacific herring and which result from a naturally occuring and widespread reservoir of virus [15]. In this instance, evolution towards a virulent state appears to have been an adaptive response of VHSV in this species.

#### Understanding the range of naturally occurring pathogens highlights the risk associated with their translocation

Understanding the phylogenetic relationships between VHSV isolates highlights the existence of major viral genotypes confined largely to different geographical areas as discussed above. The physical separation of these areas has resulted in limited geneflow and independent evolution of the viruses circulating in these regions. It stands to reason that VHS viruses in these regions have not evolved independently but have rather co-evolved with their hosts to ensure continued survival. Since viruses rely on the availabilty of hosts to avoid extinction it is perhaps not surprising to find many examples where viruses within their natural range do not cause disease in their hosts. Anthropogenic factors, including those associated with international aquaculture, have in general lead to the increased potential for VHSV viruses to be translocated outwith their naturally occurring range. An extreme example of the potential consequence of such occurrence is that of the recent series of VHS epidemics in the Great Lakes region of North America. This series of epidemics, which spread rapidly through the region and resulted in dramatic fish kills, was shown to have been caused by a virus whose natural range appeared to be marine species inhabiting the eastern coastal areas of the USA or Canada [46]. The introduction of this Genotype 1b virus into the freshwater system of the Great Lakes is thought to have originated from one of several possible sources including ballast water, baitfish translocation or by anadromous or catadromous species that can enter the Great Lakes via the St. Lawrence river [46]. The Great Lakes system is one of the largest freshwater systems in the world and is noted for its rich biodiversity [47]. The fact that VHSV virus spread rapidly through the Great Lakes system causing large scale mortality in a wide range of susceptible species, has highlighted the potential risk of translocation of viruses and exposure to previously naïve species.

#### Identification of genetic relationships is used to trace the spread of pathogens in aquaculture

Molecular epidemiology is a powerful tool, which alongside more traditional epidemiological tools has potential for tracing the origins and spread of new disease outbreaks. Such an approach relies not only on the availability of large genetic datasets relating to phylogenetically informative and defined regions of the genome but also on availability of data relating to potential epidemiological contact. The latter is often more challenging to obtain. An example of the application and potential of such tools is the recent occurrence of VHS disease in rainbow trout in the British Isles, which until this point had a history of freedom from VHS. The disease was first identified and contained on a farm in North Yorkshire, England in May 2006 and initial investigations into the likely origin of introduction proved inconclusive. Subsequent molecular epidemiological analysis of the causative virus identified a Genotype Ia virus which was very similar to isolates from Denmark and Germany circulating between 2004 and 2006 and suggested likely introduction from this region [48]. However, the lack of identification of an exact matching sequence and lack of epidemiological contact information hindered identification of a more exact source.

#### A sound knowledge of molecular epidemiology supports strategies for surveillance and further knowledge

Understanding the natural sequence variation and divergence among isolates is of fundamental importance to ensuring adoption of an appropriate surveillance regime for VHSV and in interpretation of its results. Molecular detection methods such as real-time PCR are increasing being employed in this field due to benefits including sensitivity, specificity, high throughput, ease of interpretation and ability to include appropriate controls. Molecular methods rely on the specificity of primers and probes to ensure detection of their intended targets. Targetting such methods to the required well characterised and conserved regions of the genome thus requires a thorough knowledge of molecular variation of the species. Even with this knowledge, the risk that highly specific single-plex assays such as probe-based real-time PCR could fail to detect new emerging variants of pathogens should be recognised. Such methods as developed for VHSV have to date, however, proven to be robust in detecting all known variants of the virus in known circulation [49].

Since phenotypic properties of viruses may in some cases be consistent with genetic origin, selecting isolates representative of the different genotypes can be a sensible strategy for further research into establishing their biological properties. Previous work on VHSV has investigated the consistency of pathogenic properties of different genotypes for different species [45,50]. Selection of representative strains for selection on this basis also ensures that the widest knowledge of the properties of the species as a whole can be obtained. Alternatively, other studies have specifically selected virus isolates of known genetic similarity that were expected to display different pathogenic properties based on their history of origin. In this way, very similar isolates with only 5 amino acid substitutions were identified, which displayed differing virulence for rainbow trout [51]. Such work provides an important basis for resolving those epitopes responsible for conferring the virulent phenotype, which in turn can lead to improved methods for disease control.

### Salmonid alphavirus (SAV)

#### Overview

Salmonid alphaviruses are responsible for salmon pancreas disease (SPD) and sleeping disease (SD) conditions, primarily of farmed Atlantic salmon and rainbow trout, respectively. Clinical signs associated with SPD include abnormal swimming behaviour and lack of appetite, while characteristic histopathological signs include severe degeneration of exocrine pancreas, cardiomyopathy and skeletal myopathy [52]. SD involves similar lesions but often manifests itself as fish lying on their side or "sleeping" as a consequence of extensive necrosis of skeletal red muscle [53]. Until recently, the presence of SAV has not been reported outside of salmonid aquaculture, where it is responsible for significant economic loss, principally in Western Europe.

#### Molecular epidemiology facilitates the taxonomic classification of SAV and provides a basis for understanding the relationship between isolates

Molecular analyses of salmonid alphaviruses have recently demonstrated that they represent a group of viruses within the genus *alphaviridae *in the family *Togaviridae*, and differ markedly from the previously established New World viruses of Venezuelan equine encephalitis virus (VEEV) and eastern equine encephalitis virus and the Old World viruses of Aura and Sindbis virus [54]. Salmon pancreas disease virus (SPDV) has been officially recognised as a species by the International Committee for the Taxonomy of Viruses (ICTV) which includes isolates causing both SD and SPD in rainbow trout and Atlantic salmon [55].

Classification in this way can give clues as to the potential biological properties and functions of salmonid alphaviruses based on the established properties of their better studied relatives. Interestingly, most alphavirus lifecycles involve an obligate, commonly arthropod intermediate host. Such an intermediate host does not appear to be a requirement of salmonid alphavirus transmission since these viruses have been shown to efficiently transmit horizontally via direct water-borne transmission [56]. Whether arthropods (e.g. sealice) have a potential role on the epizootiology of salmonid alphaviruses remains an interesting research question.

**Figure 3:**
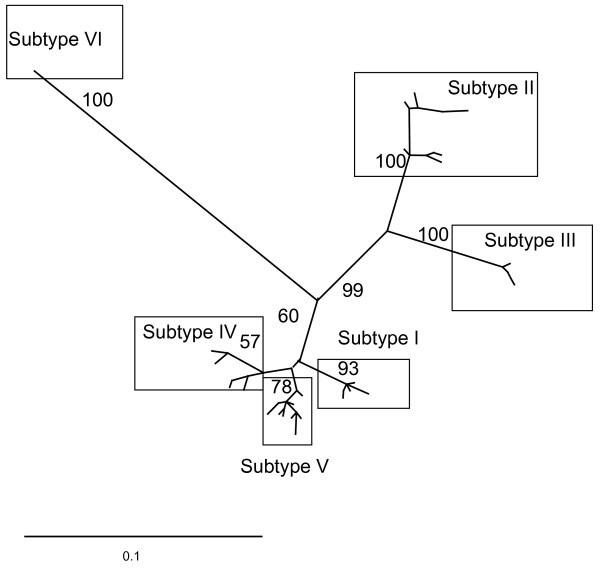
**Overview of the genetic relationships between the main recognized genotypes of SAV based on a dataset of partial E2 sequence data (298nt)**. Publically available sequences derived from isolates from each subtype recognized to date were imported into Bioedit version 7.0.5.3 [76] and a multiple alignment performed using Clustal X [77]. Duplicate sequences were removed and the final alignment consisted of 36 unique sequences representing a total of 83 isolates and spanned a region of 298nt of the open reading frame of SAV E2 gene. The phylogenetic relationship among SAV isolates was inferred using the method described in the legend of Figure 2.

The most comprehensive molecular epidemiological analysis of the genetic relationships within the salmonid alphaviruses was recently conducted by Fringuelli et al. [57]. This relationship is illustrated in Figure 3. The existence of a total of six discrete and identifiable subtypes has been suggested based on analysis of partial E2 and nsP3 sequences. Subtypes I, IV and V are genetically very closely related and include isolates from Atlantic salmon farms in the British Isles responsible for salmonid pancreas disease (SPD). Subtype VI included a single isolate from Ireland. The disease in Norway has to date been exclusively associated with a different genetic group of viruses belonging to subtype III. Subtype II isolates include those which have caused sleeping disease (SD) in rainbow trout farms from Europe. While viruses responsible for SPD in Atlantic salmon and SD in rainbow trout generally fall within different genetic subgroups there are exceptions. This fact, coupled to their close genetic similarity supports the taxonomic designation of the salmonid alphaviruses as a single species.

#### Understanding genetic relationships provides an insight into the origins of SAV in aquaculture

The monophyletic nature of the freshwater rainbow trout subtype of SAV (Subtype II, Figure 3) has been interpreted to indicate a single introduction and subsequent dissemination of SAV within this industry. This, based on current knowledge might be expected to have occurred from a salmonid source. Recent information, however, derived from the screening of marine fish species by real-time PCR in areas remote from aquaculture, has indicated the presence of SAV RNA in marine species including common dab (*Limanda limanda*) and plaice (*Pleuronectes platessa*) [58]. The identification of subtype V SAV sequences associated with these detections suggests that, as in the case for VHSV, marine reservoirs of SAV might exist. Whilst the exact implications of this remain unclear, it is possible that the origins of SAV in freshwater rainbow trout may have been from a similar marine source or indeed, import via contact with infected Atlantic salmon. It is interesting to speculate as to whether in time a more extensive marine reservoir of SAV will become apparent, as has been shown for VHSV, and whether this might suggest independent import of SAV into Norwegian and UK aquaculture respectively.

#### Identification of genetic relationships is used to trace the spread of pathogens in aquaculture

Molecular epidemiology has been applied to the study of SAV and has provided evidence to support farm to farm transmission in both Scotland and Ireland where clusters of identical sequences from isolates from different farms located within the same bodies of water have been demonstrated [57]. Furthermore, the fact that sequences from one cluster of outbreaks in the West of Ireland was associated with a subtype of virus distinct to that prevalent in the region of origin of these fish supports horizontal rather than vertical transmission as the predominant source of infection. Horizontal transmission can occur directly through the water or via fomites, and SAV has been shown to have a mimimum half-life of 5.7 days in seawater at 10°C [59]. Having said this, understanding the epizootiology of SAV is complex for a number of reasons. Firstly, the disease is not notifiable in the UK, meaning than information on disease occurrence and characterisation of responsible agents is often lacking. Secondly, the virus appears to have a relatively low rate of evolution and identical viral sequences have been reported to be detected over extended periods of time within single populations, within farms and between different farms. This makes interpretation of epidemiological links difficult. A clearer explanation has, however, been identified for the import of sleeping disease into the UK, since evidence of French common ancestry of contemporary isolates circulating in both France and Scotland/England supports epidemiological evidence that import was associated with the processing of fish imported from France in 2002 [57,60,61].

#### A sound knowledge of molecular epidemiology supports strategies for surveillance and further knowledge

Knowledge of molecular epidemiology and genetic diversity of SAV has led to the development of sensitive and specific molecular diagnostic methods [62] capable of detecting the range of reported SAV subtypes. Further, these methods are capable of subtype discrimination and might prove useful in rapid screening of isolates to highlight translocation of new subtypes to new geographic areas. Genotypic differentiation has again been used to identify isolates for further detailed research, in particular to understand the clinical presentation of disease following infection with different genotypes [63]. Recent work developing reverse genetics approaches to study SAV [64] offers great potential in realising the biological significance of the genetic differences between strains. Such work has important implications for disease surveillance programmes in addition to contributing to a deeper understanding of the biology of the causative agent and thus how to apply practical disease mitigation control strategy.

### Infectious salmon anaemia virus (ISAV)

#### Overview

Infectious salmon anaemia (ISA) is a disease of farmed Atlantic salmon that has been responsible for extensive losses in all major regions where this species is farmed including, Norway, Canada, Scotland, the Faroe Islands and Chile. The disease is characterised by severe anaemia, ascites and haemorrhagic liver necrosis and congestion [65]. While continued disease problems occur in Norway, Canada and Chile, the disease has successfully been eradicated from both Scotland and the Faroes. In the latter case, however, this was only achieved at the expense of collapse of the entire industry.

#### Molecular epidemiology facilitates the taxonomic classification of ISAV and provides a basis for understanding the relationship between isolates

**Figure 4:**
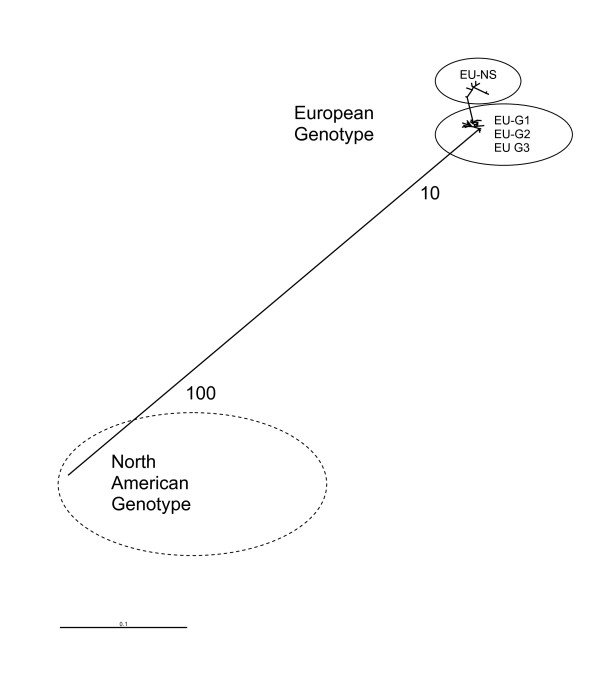
**Overview of the genetic relationships between the two main identified genotypes of ISAV**. Publically available ISAV sequences were imported into Bioedit version 7.0.5.3 [76]and a multiple alignment performed using Clustal X [77]. Identical sequences were identified and only a single representative of each sequence type retained in the dataset to reduce subsequent analytical bias. The final alignment consisted of 38 unique sequences spanning a region of 987 (positions 25-1012 with respect to the open reading frame of ISAV segment 6). The phylogenetic relationship among ISAV isolates was inferred using the method described in the legend of Figure 2.

Molecular epidemiological study has made a significant contribution to our understanding of ISAV. Following identification and initial characterisation, the virus was recognised as an orthomyxovirus [66], and ultimately assigned to its own genus. Orthomyxoviruses such as influenza viruses are characterised by a high potential for adaptation via a repertoire of mechanisms for genetic change including mutation, reassortment and recombination. Indeed, these properties have all been demonstrated for ISAV within aquaculture [67]. Relationships among ISAV isolates have been explored most extensively on the basis of their surface protein coding genes, namely the haemagglutinin esterase (HE) and fusion (F) protein genes [68,69]. Though some complicated subdivisions of ISAV subtypes have been proposed, these analyses have revealed the same general pattern of existence of single major European and North American genotypes of ISAV (Figure 4). Within the European genotype of ISAV, different subtypes have been proposed to classify isolates from Europe (EUG1, EUG2 and EUG3), and some isolates from North America (EU-NS) which appear to share a more recent common ancestor with European ISAV than with the true North American ISAV genotype.

#### Understanding genetic relationships provides an insight into the origins of ISAV in aquaculture

North American and European evolutionary lineages of ISAV appear to have been present and diverged long before the development of commercial Atlantic salmon aquaculture [70]. Interestingly, all pathogenic variants of ISAV associated with disease outbreaks are characterised by the occurrence of a sequence deletion within the so-called highly polymorphic region (HPR), with respect to a putative ancestral form of the HE gene [69]. This putative ancestral form is designated HPR0 and its presence has been detected in healthy farmed and wild fish in all the major regions of Atlantic salmon production worldwide including, Norway [12,69], Scotland [71,72], Canada [73], Chile [68] and the Faroe Islands (Dr Christiansen, personal communication). Molecular epidemiological evidence supports the suggestion that pathogenic ISAV which causes disease in fish farms likely originates from a reservoir of the ISAV-HPR0 variant since a wide variety of HPR0 sequences reflecting the broad diversity of virulent isolates have been identified and are distributed throughout the tree depicted in Figure 4. The mechanisms governing adaptation of the ISAV-HPR0 variant in aquaculture are, however, poorly understood. The fact that new cases of ISA which have emerged in countries previously free of the disease, however, does suggest that a lack of synchronous fallowing and thus provision of opportunity for pathogens to persist over long time periods may contribute to the risk of emergence of disease. In this instance a better understanding of the potential risk factors in emergence of ISA disease has contributed to practical fish health policy aimed at mitigating such risks. Such solutions include the creation of management areas, which amongst other measures aim to achieve periods of synchronous fallowing in order to limit opportunity for pathogens, including ISAV to reside within production systems.

#### Identification of genetic relationships is used to trace the spread of pathogens in aquaculture

Molecular epidemiogical study has been applied to understanding the appearance of ISA in Chile [68], which has caused a significant decline in production. Molecular evidence in this instance pointed strongly towards a Norwegian source, with vertical or trans-generational transmission being highlighted as a plausible explanation, based on the significant and historical import of fertilised eggs into Chile from this area. Such knowledge has raised concerns over the future risk of import of disease in association with ova and has lead to an application of biosecurity measures aimed at guarding against such import. This includes broodstock testing regimes based on molecular detection, accompanied by destruction of ova originating from positive-testing parents and the development of protocols for the disinfection of imported egg products. Whether true vertical transmission of ISAV (ie the internal transmission of virus within the gametes) occurs in ISAV remains somewhat controversial though it has been suggested [69]. It seems clear from evidence in Chile that transgenerational transmission is highly likely to have occurred. Whether this represents true vertical transmission or resulted from inadequate disinfection of eggs remains uncertain. In this case, the application of molecular epidemiological study has identified the probable origin of import of ISAV into Chile, thus highlighting a potential risk that can be mitigated based on the implementation and maintenance of appropriate biosecurity measures. Since horizontal transmission remains the main mechanism for dissemination of ISAV where it is present, the major challenge now faced, is to apply molecular epidemiological tools to understand and control the spread of ISA within Chile.

#### A sound knowledge of molecular epidemiology supports strategies for surveillance and further knowledge

Molecular detection methodologies have been developed to cover the broad range of ISAV viruses in global circulation [74], providing confidence that the pathogen can be accurately detected. Information on the existence of the ISAV-HPR0 variant has been invaluable in interpreting the results of molecular diagnostic tests which currently do not differentiate between this and other variants of the virus. The fact that the ISAV-HPR0 variant has been shown to be present, even in territories with freedom of ISA disease, suggests a clear distinction in risk associated with the presence of ISAV-HPR0 and other disease causing variants. Differentiation of viruses with different biological properties such as HPR0, also provides the basis for developing understanding of the genetic determinants of biological phenotype. Such knowledge is fundamental to managing pathogen-associated risk in open aquaculture systems.

### Future perspectives and challenges

The application of molecular epidemiology to the study of fish viruses has to date largely focussed on the use of descriptive techniques and interpretation of genetic relationships has often been hampered by a lack of associated analytical epidemiological information. The development of next generation sequencing technology promises a revolution in the ability to generate sequence data and thus information of potential epidemiological relevance. Descriptive molecular data alone is, however, inadequate in tracing pathogen spread, especially when variation is limited and evolution does not occur in a clock-like or regular fashion. The ability of molecular epidemiology to fulfil its potential to translate complex disease pathways into relevant fish health policy is thus unlikely to be limited by the generation of descriptive molecular markers. More likely, full realisation of the potential to better explain viral transmission pathways will be dependent on the ability to assimilate and analyse knowledge from a range of more traditional information sources. The development of methods to systematically record and share such epidemiologically important information thus represents a major challenge for fish health professionals in making the best future use of molecular data in supporting fish health policy and disease control.

Making best use of this generated data to better understand the molecular basis of virulence also remains an important area for future research. Significant progress has been made in developing reverse genetic approaches for fish viruses (for review see [75]) which offer great potential for better understanding viral virulence and thus the risk factors which may contribute to the emergence of new pathogens.

Despite the limitations in available knowledge, molecular epidemiology has led to an improved understanding of the origins and spread of viruses in aquaculture and wild fish, which in turn has lead to significant practical improvements in disease management strategies and policy. A common theme in the examples explored seems to be the prevalence of probably benign viruses in the environment, which undergo an adaptation in association with aquaculture, where they adopt a pathogenic lifestyle. Major challenges for sustainable aquaculture are to manage the risk associated with such occurrence by reducing the opportunity for pathogens to reside and thus potentially adapt within aquaculture systems, coupled to their rapid identification and containment following disease emergence. Both these factors can be facilitated through implementation of biosecurity measures and the use of synchonous fallowing strategies to break potential long term cycles of infection and ensure rapid and appropriate containment of disease in discrete management areas. The implementation of surveillance programs based on the best available molecular epidemiological information offers great potential to support such measures in further developing a healthy and sustainable aquaculture industry that is necessary to satisfy an increasing world demand for cultured fish products.

## Competing interests

The author declares that they have no competing interests.
